# Associations of multi-human papillomavirus infections with expression of p16 in a cohort of women who underwent colposcopy: a retrospective study of 5165 patients

**DOI:** 10.3389/fonc.2023.1265726

**Published:** 2023-10-26

**Authors:** Yulong Zhang, Haibo Li, Xiaowen Li, Zelong Li, Qianru You, Hanwen Liu, Zhiyan Zhao, Yanzhao Su, Xiangqin Zheng, Yusha Chen, Jiancui Chen, Huan Yi

**Affiliations:** ^1^ Department of Gynecology, Fujian Maternity and Child Health Hospital College of Clinical Medical for Obstetrics & Gynecology and Pediatrics, Fujian Medical University, Fuzhou, China; ^2^ Division of Birth Cohort Study, Fujian Maternity and Child Health Hospital, College of Clinical Medicine for Obstetrics & Gynecology and Pediatrics, Fujian Medical University, Fuzhou, China; ^3^ Integrated Biology, University of California, Berkeley, Berkeley, CA, United States; ^4^ Cervical Disease Diagnosis and Treatment Health Center, Fujian Maternity and Child Health Hospital College of Clinical Medical for Obstetrics & Gynecology and Pediatrics, Fujian Medical University, Fuzhou, China

**Keywords:** HPV, cervical cancer, p16, cervical lesions, retrospective study

## Abstract

**Objective:**

Investigate HPV types in cervical specimens, their correlation with p16 expression in lesions, and diagnostic value for cervical lesions. Enhance clinical diagnosis reliability.

**Methods:**

Retrospective cross-sectional study at Fujian Maternity and Child Health Hospital’s Cervical Disease Center (Jun 2019-Dec 2021). Patients with abnormal cervical screening underwent colposcopy and conization. Pathological diagnosis based on colposcopy, cervical biopsy, ECC, and conization. Analyzed HPV genotyping (18 HR-HPV, 5 LR-HPV) and p16 expression correlation. Statistical analysis used R software.

**Results:**

he expression of p16 is significantly associated with the infection of high-risk HPV types, such as 16, 33, 52, and 58, with an increased risk of 1.4 times or higher (OR=1.91, 3.14, 1.40, and 1.78, respectively). The risk of p16 expression increased 4-fold for multiple high-risk HPV types [adjusted OR (95% CI) = 4 (2.92~5.5), P-value <0.001]. Compared to the p16(-) group, the p16(+) group had a higher association with cervical lesions worse than HSIL (High-grade Squamous Intraepithelial Lesions).In the group with multiple Human Papillomavirus Infections with types 16, 33, 52, and 58, the risk of cervical lesions worse than HSIL increased by up to 660-fold compared to the negative group (adjusted OR=660.62, 95% CI: 91.39~4775.53, P<0.001), indicating that this combination of HPV types posed the greatest risk for cervical lesions above HSIL.

**Conclusions:**

p16 plays a crucial role in cervical lesion progression, linked to high-risk HPV. Combining p16 with HPV screening improves cervical cancer detection. Studying multiple HPV infections will enhance prevention and management.

## 1 Background

Cervical cancer is one of the most common gynecological malignancies worldwide, with the highest incidence among malignant neoplasms of the female reproductive system, only second to breast cancer (1). At present, cervical cancer causes up to 30,000 deaths of women in China every year, which poses a huge threat to women’s health in the country (2).

The development of cervical cancer is a long-term and continuous process of tumor progression, which includes cytological abnormalities, low-grade squamous intraepithelial lesion (LSIL), high-grade squamous intraepithelial lesion (HSIL), and finally, carcinogenesis. This process requires the involvement of multiple pathogenic factors, multiple oncogenes, and occurs through a series of steps (3, 4). One crucial factor in the development of cervical cancer is persistent infection with human papillomavirus (HPV). The World Health Organization (WHO) has listed cervical cancer as the first most common cancer caused by HPV infection (5).

Currently, HPV-DNA detection is the primary screening method for cervical cancer in China. However, it has its limitations, including high sensitivity and low specificity due to the influence of various factors in both the host and the virus (6). Especially for precancerous lesions, HPV-DNA testing is only a qualitative test, which cannot classify the severity of lesions nor distinguish between transient and persistent infections. As a result, it cannot guarantee the accuracy of cancer diagnoses (6, 7). There was also research revealed the importance of the HPV mRNA test to define how severe is a cervical lesion, more research is needed to prove (8).

To improve the accuracy of cervical cancer detection and prognosis, researchers have been investigating the role of p16, a tumor suppressor gene involved in the progression of uterine cervical lesions (9). The p16 protein, produced by this gene, has been found to inhibit the cell cycle, thereby negatively regulating cell growth, and controlling cell hyperproliferation. Dysfunctional pathways resulting from aberrant p16 protein expression may induce cervical intraepithelial neoplasia (CIN) and influence the occurrence and development of cervical cancer (10–12).

However, while the significance of p16 in cervical cancer progression has been studied, there is still a lack of research on its interaction with different HPV infection genotypes (13). As a result, the relationship between p16 expression and cervical lesions remains unclear, and the potential value of combining HPV detection with p16 testing in differentiating cervical lesions needs further exploration.

In this study, we conducted a retrospective analysis of patients with cervical lesions using histopathology as the standard for diagnoses (14). All patients underwent HPV typing and p16 expression testing. The main objective was to evaluate the diagnostic significance of HPV typing and p16 detection alone or in combination for cervical lesions, aiming to provide a more reliable clinical diagnosis method. This approach would help avoid overtreatment and reduce the rate of misdiagnosis in patients with mild lesions confirmed by postoperative pathology (14).

## 2 Materials and methods

### 2.1 Study population

**Figure 1:**
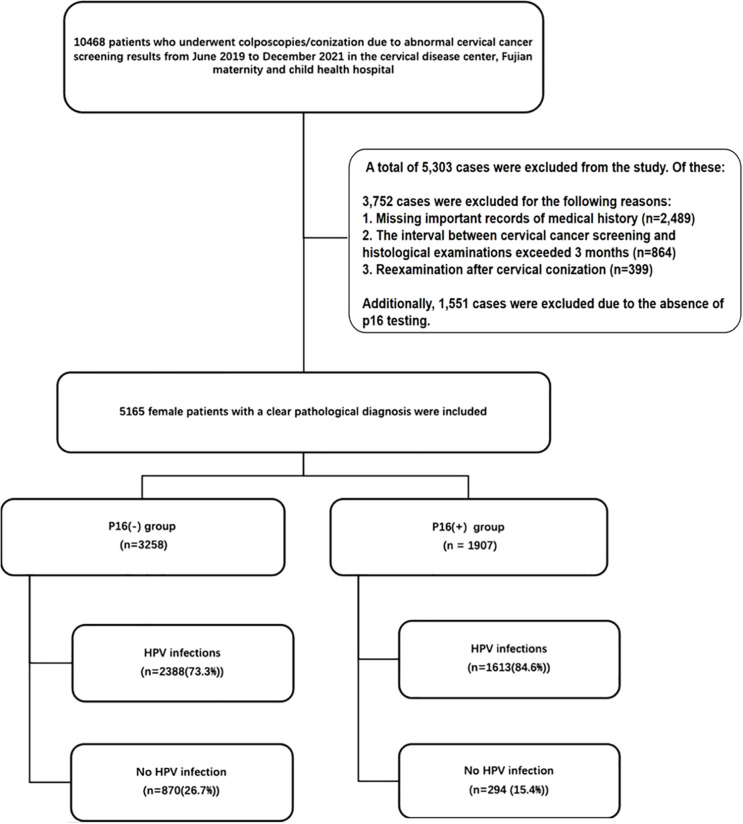
Out of a total of 10,468 patients who underwent colposcopies/conization due to abnormal cervical cancer screening results from June 2019 to December 2021, 5,303 cases were excluded. Of these, 3,752 cases were excluded for the following reasons: 1. Missing important records of medical history (n=2,489). 2. The interval between cervical cancer screening and histological examinations exceeded 3 months (n=864). 3. Reexamination after cervical conization (n=399). Additionally, 1,551 cases were excluded due to the absence of p16 testing. A total of 5,165 female patients with clear pathological diagnoses were included in the final analysis. HPV, human papillomavirus.

This cross-sectional study included patients who underwent colposcopy and conization due to abnormal cervical cancer screening results at the cervical disease center of Fujian Maternity and Child Health Hospital from June 2019 to December 2021. Cervical cancer screening involved ThinPrep Cytology Test (TCT) and/or HPV genotyping. Abnormal cytology results were defined as Atypical Squamous Cells of Undetermined Significance (ASC-US), Low-grade Squamous Intraepithelial Lesion (LSIL), High-grade Squamous Intraepithelial Lesion (HSIL), Atypical Glandular Cells (AGC), Endocervical Adenocarcinoma *in situ* (AIS), Squamous Cell Carcinoma (SCC), and Adenocarcinoma. The interval between cervical cancer screening and histological examination was less than 3 months. Clinical information, including age, gravidity, parity, HPV genotypes, and cervical pathology, was extracted from the department’s medical records (**Figure 1**).

The study was conducted in compliance with the Declaration of Helsinki (as revised in 2013) and was approved by the Ethics Committee of Fujian Maternity and Child Health Hospital, Affiliated Hospital of Fujian Medical University (2023KY038). Due to the retrospective nature of the study, informed consent was exempted.

### 2.2 HPV Genotyping

PCR-RDB HPV genotyping (Yaneng Biotech) was performed to identify 18 genotypes of high-risk HPV (HR-HPV): HPV-16, 18, 31, 33, 35, 39, 45, 51, 52, 53, 56, 58, 59, 66, 68, 73, 82, and 83, as well as 5 types of low-risk HPV (LR-HPV): HPV-6, 11, 42, 43, and 81.

### 2.3 Pathological diagnosis

Colposcopy referrals were based on the ASCCP guidelines (10). All patients underwent colposcopy and cervical biopsy. Additionally, patients with HPV-16 and 18 infections, AGC/AIS/HSIL cytology, and type 3 cervical transformation zone underwent endocervical curettage (ECC). Cervical cone resection was performed in cases with liquid-based cytology results indicating HSIL, AGC-FN (atypical glandular cell, favor neoplastic), AIS, or cervical pathological biopsy and ECC results indicating CIN2-3 (cervical intraepithelial neoplasia 2-3). Two blinded senior pathologists independently performed the pathological evaluation of cervical biopsies, ECC, and conization tissues. Standard haematoxylin-eosin stain was used in this study, standard H&E protocol allows visualization of tissue morphology by imparting blue-stained nuclei and pink-stained cytoplasm/connective tissue. It is the routine stain for histopathology, providing an overview of tissue architecture and cytology.

The final pathological diagnosis was determined using the most severe result among evaluations of cervical biopsies, ECC, and conization tissues. The histologic endpoints were defined according to the 2014 WHO classification of tumors of the female reproductive organs (4th Edition) (11) and Lower Anogenital Squamous Terminology (LAST) recommendations as follows (12): Normal cervix; LSIL, which includes CIN1 and p16 negative CIN2; HSIL, including p16 positive CIN2 and CIN3; AIS; invasive cervical cancer. Furthermore, HSIL, AIS, and invasive cervical cancer were classified as HSIL+.

### 2.4 Procedure for colposcopic examination and immunocytochemical staining

The Leisegang D-10625, Model1DS Ur Nr 55764, Colposcope from Berlin, Germany, was used for cervix examination. After exposing the cervix using an appropriately sized Cusco’s speculum, the vulva, vagina, and cervix were examined before the application of 3% acetic acid solution for each patient. Colposcopic abnormalities were classified as normal, abnormal, or unsatisfactory. Biopsies were taken from abnormal areas using punch cervical biopsy tissue forceps. The cervical specimens were processed in the histopathology laboratory, and a histopathologist blinded to the HPV status of the participants performed the diagnosis. Immunocytochemical staining was performed using the P16/Ki67 double staining kit on each cervical specimens. Experimental operations were strictly in accordance with the kit instructions and the technical instructions for double staining of cervical cells, and two experienced pathologists conducted and interpreted the double staining of cervical epithelial cell.

### 2.5 Statistical analysis

Categorical variables were presented as frequencies (percentages), and statistical analyses were performed using R software and its packages (Open Access, Version 4.0.2). Descriptive statistics showed mean ± standard deviation for continuous variables, while frequency and percentage were used for categorical variables. The statistical differences among p16 status for clinical characteristics were tested with t-tests for continuous variables and Chi-square tests for categorical variables. Univariate and multivariate logistic regression analyses, adjusting for age, gravidity, parity, and pregnancy, were used to determine the association between multiple HPV infections and cervical lesions. Two-tailed P-values less than 0.05 were considered statistically significant.

## 3 Results

### 3.1 Characteristics of patients

**Figure 2:**
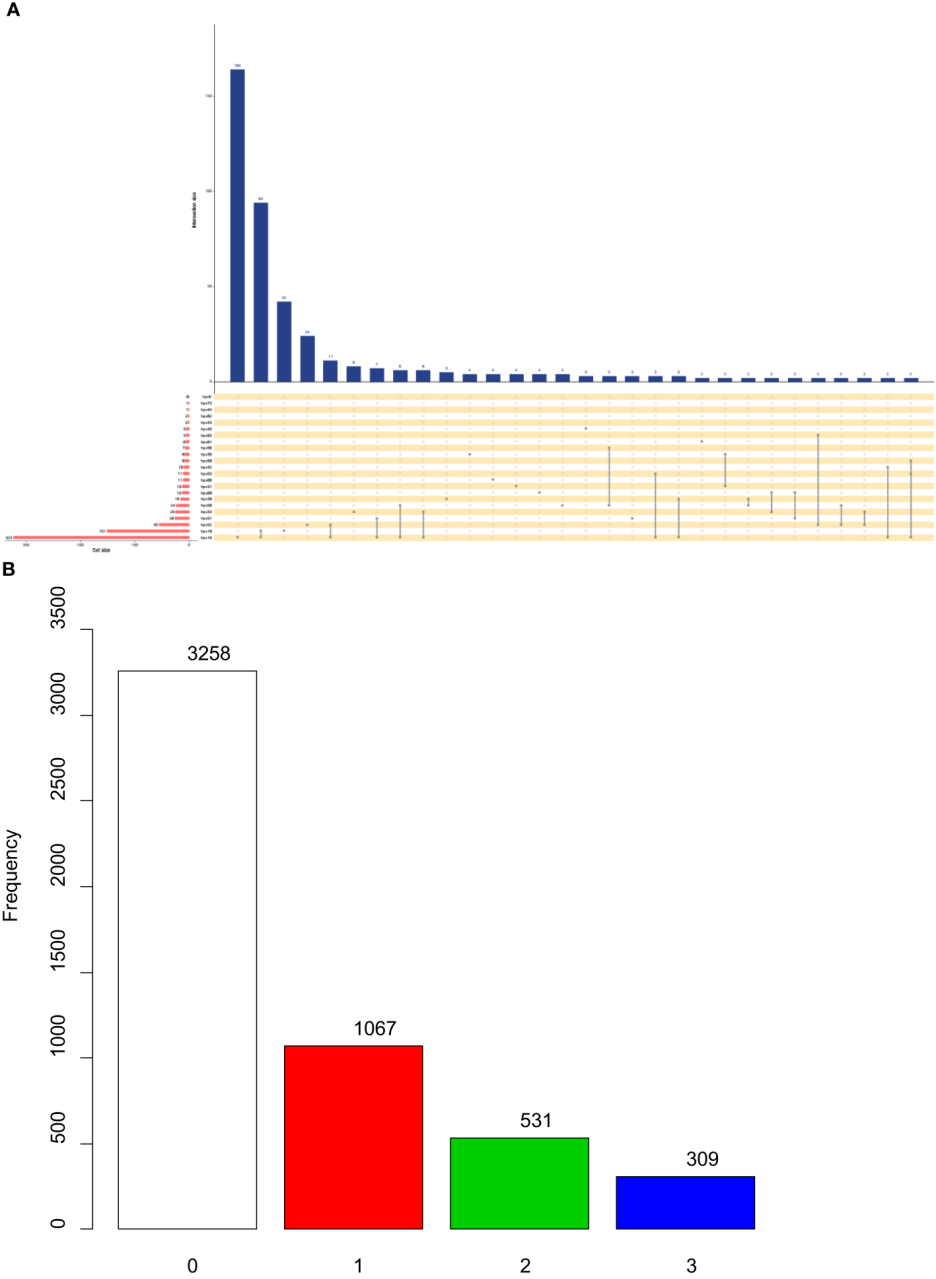
Upset plots of the intersections of HPV genotype **(A)** and different expression level of p16 **(B)**. Each row corresponds to a set of infection genotype(s), and the bar chart on the left demonstrates the size of each set. Each column corresponds to a possible intersection: the filled-in cells show which set is a part of an intersection.

The analysis included a total of 5165 female patients with definitive pathological diagnoses. Among them, there were 3258 cases with p16(-) and 1907 cases with p16(+). The mean age of patients with p16(+) was significantly older than that of patients with p16(-) (42.5 ± 11.1 vs. 39.4 ± 10.9, p<0.001). The prevalence of HPV infection was 73.3% (n=2388) in patients with p16(-), whereas it was 84.6% (n=1613) in patients with p16(+) (P<0.001). P16(+) was associated with the infection of high-risk HPV types 16, 33, 52, 56, 58, and low-risk HPV type 81 (P<0.05) (**Table 1**). **Figure 2** shows the intersections of the HPV genotype. HPV genotypes 16, 18, 52, 51 and 33 had the most frequent infections, and there was coinfection (**Figure 2**).

**Table 1:** Characteristics of the study patients.

Variables	Total (n = 5165)	p16- (n = 3258)	p16+ (n = 1907)	p
Ages, Mean ± SD	41.3 ± 11.1	39.4 ± 10.9	42.5 ± 11.1	< 0.001
gestation, n (%)				0.022
0	406 (7.9)	230 (7.1)	176 (9.2)	
1	797 (15.4)	493 (15.1)	304 (15.9)	
2	1357 (26.3)	851 (26.1)	506 (26.5)	
3	1165 (22.6)	765 (23.5)	400 (21)	
≥4	1440 (27.9)	919 (28.2)	521 (27.3)	
parity, n (%)				0.001
0	730 (14.1)	417 (12.8)	313 (16.4)	
1	1862 (36.1)	1220 (37.4)	642 (33.7)	
2	1929 (37.3)	1211 (37.2)	718 (37.7)	
≥3	644 (12.5)	410 (12.6)	234 (12.3)	
hpv16, n (%)				< 0.001
0	3507 (67.9)	2398 (73.6)	1109 (58.2)	
1	1658 (32.1)	860 (26.4)	798 (41.8)	
hpv18, n (%)				0.179
0	4047 (78.4)	2572 (78.9)	1475 (77.3)	
1	1118 (21.6)	686 (21.1)	432 (22.7)	
hpv31, n (%)				0.916
0	5050 (97.8)	3186 (97.8)	1864 (97.7)	
1	115 (2.2)	72 (2.2)	43 (2.3)	
hpv33, n (%)				< 0.001
0	5036 (97.5)	3210 (98.5)	1826 (95.8)	
1	129 (2.5)	48 (1.5)	81 (4.2)	
hpv35, n (%)				0.526
0	5096 (98.7)	3217 (98.7)	1879 (98.5)	
1	69 (1.3)	41 (1.3)	28 (1.5)	
hpv39, n (%)				0.789
0	5020 (97.2)	3165 (97.1)	1855 (97.3)	
1	145 (2.8)	93 (2.9)	52 (2.7)	
hpv45, n (%)				0.617
0	5103 (98.8)	3217 (98.7)	1886 (98.9)	
1	62 (1.2)	41 (1.3)	21 (1.1)	
hpv51, n (%)				0.414
0	4916 (95.2)	3107 (95.4)	1809 (94.9)	
1	249 (4.8)	151 (4.6)	98 (5.1)	
hpv52, n (%)				< 0.001
0	4456 (86.3)	2857 (87.7)	1599 (83.8)	
1	709 (13.7)	401 (12.3)	308 (16.2)	
hpv53, n (%)				0.242
0	4894 (94.8)	3078 (94.5)	1816 (95.2)	
1	271 (5.2)	180 (5.5)	91 (4.8)	
hpv56, n (%)				0.017
0	5029 (97.4)	3159 (97)	1870 (98.1)	
1	136 (2.6)	99 (3)	37 (1.9)	
hpv58, n (%)				< 0.001
0	4835 (93.6)	3091 (94.9)	1744 (91.5)	
1	330 (6.4)	167 (5.1)	163 (8.5)	
hpv59, n (%)				0.24
0	5031 (97.4)	3167 (97.2)	1864 (97.7)	
1	134 (2.6)	91 (2.8)	43 (2.3)	
hpv66, n (%)				0.264
0	5053 (97.8)	3193 (98)	1860 (97.5)	
1	112 (2.2)	65 (2)	47 (2.5)	
hpv68, n (%)				0.838
0	5021 (97.2)	3166 (97.2)	1855 (97.3)	
1	144 (2.8)	92 (2.8)	52 (2.7)	
hpv73, n (%)				0.281
0	5142 (99.6)	3241 (99.5)	1901 (99.7)	
1	23 (0.4)	17 (0.5)	6 (0.3)	
hpv82, n (%)				0.383
0	5131 (99.3)	3239 (99.4)	1892 (99.2)	
1	34 (0.7)	19 (0.6)	15 (0.8)	
hpv42, n (%)				0.066
0	5029 (97.4)	3162 (97.1)	1867 (97.9)	
1	136 (2.6)	96 (2.9)	40 (2.1)	
hpv43, n (%)				0.403
0	5082 (98.4)	3202 (98.3)	1880 (98.6)	
1	83 (1.6)	56 (1.7)	27 (1.4)	
hpv44, n (%)				0.518
0	5142 (99.6)	3242 (99.5)	1900 (99.6)	
1	23 (0.4)	16 (0.5)	7 (0.4)	
hpv81, n (%)				< 0.001
0	5021 (97.2)	3141 (96.4)	1880 (98.6)	
1	144 (2.8)	117 (3.6)	27 (1.4)	
hpv83, n (%)				0.39
0	5083 (98.4)	3210 (98.5)	1873 (98.2)	
1	82 (1.6)	48 (1.5)	34 (1.8)	
hpv, n (%)				< 0.001
0	1164 (22.5)	870 (26.7)	294 (15.4)	
1	4001 (77.5)	2388 (73.3)	1613 (84.6)	

Each row corresponds to a set of infection genotype(s), and the bar chart on the left demonstrates the size of each set. Each column corresponds to a possible intersection: the filled-in cells show which set is a part of an intersection.

### 3.2 Association between different HPV genotype and p16

**Figure 3:**
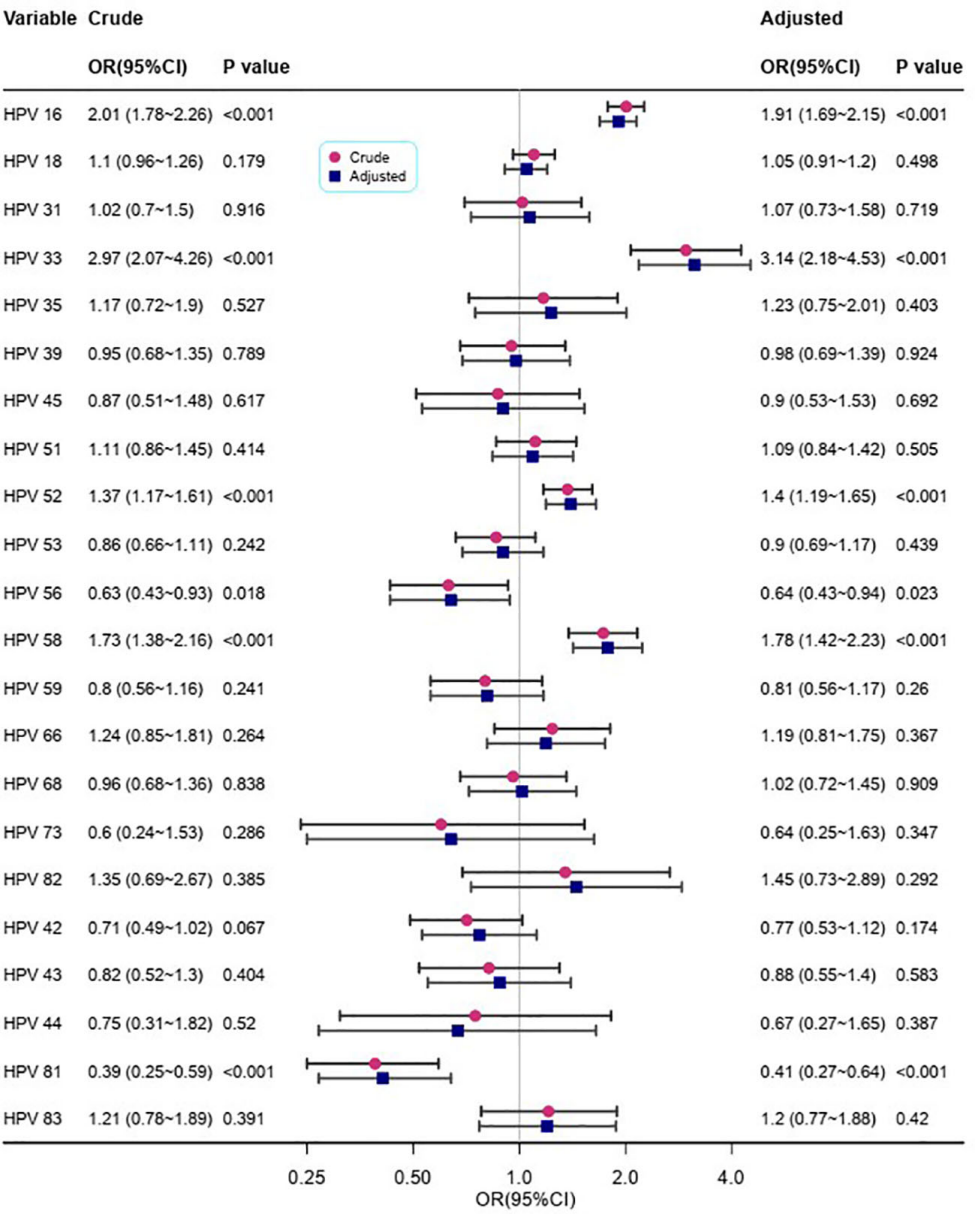
The relationships between different HPV genotypes and p16.

**Figure 3** depicts the relationship between different HPV genotypes and p16 expression. In the crude models, high-risk HPV types 16, 33, 52, 56, 58, and 81 showed a significant correlation with p16 expression, whereas a negative relationship was observed for HPV type 18. After adjusting for confounding factors, the results remained consistent with the univariate analysis. Infection with high-risk HPV types increased the risk of p16(+) by approximately 1.4 times or higher (OR=1.91, 3.14, 1.40, and 1.78, respectively).

Models adjusted for age, gravidity, parity, and cervical histology.

### 3.3 Association between multiple HPV infections and p16

**Table 2** presents the results of univariate and multivariate logistic regression analyses of multiple HPV infections and p16 expression. The highest incidence of p16(+) was identified in individuals infected with HPV33+ and multiple high-risk HPV infections (MH-HPV+). Moreover, there was an increased risk of p16(+) for HPV genotypes 16, 33, 52, and 58 alone, as well as for multiple high-risk HPV infections.

**Table 2:** Univariate and multivariate logistic regression analyses of HPV infection patterns and p16.

Variables	Total	Event (%)	Crude		Adjusted	
			OR (95%CI)	P value	OR (95%CI)	P value
negative	1164	294 (25.3)	1(Ref)		1(Ref)	
HPV16+	820	399 (48.7)	2.8 (2.32~3.39)	<0.001	2.65 (2.19~3.22)	<0.001
HPV33+	65	39 (60)	4.44 (2.66~7.42)	<0.001	4.38 (2.61~7.36)	<0.001
HPV52+	390	163 (41.8)	2.12 (1.67~2.7)	<0.001	2.08 (1.63~2.66)	<0.001
HPV58+	160	75 (46.9)	2.61 (1.86~3.66)	<0.001	2.62 (1.86~3.68)	<0.001
MH-HPV+	195	112 (57.4)	3.99 (2.92~5.46)	<0.001	4 (2.92~5.5)	<0.001
OtherHPV+	1375	379 (27.6)	1.13 (0.94~1.34)	0.19	1.11 (0.93~1.33)	0.249
MultiOtherHPV+	996	446 (44.8)	2.4 (2~2.88)	<0.001	2.31 (1.92~2.77)	<0.001

Models adjusted for age, gravidity, parity, and cervical histology.

MH-HPV+, Multiple HPV16/33/52/58 infection. OtherHPV+, Single HPV infection with genotype other than HPV16, 33, 52, and 58. MultiOtherHPV+, Multiple HPV infection with genotype other than HPV16, 33, 52, and 58.

Specifically, the risk of p16(+) increased 4.38-fold when infected with HPV33 alone [adjusted OR (95% CI) = 4.38 (2.617.36), P < 0.001], and 4-fold when infected with multiple high-risk HPV genotypes [adjusted OR (95% CI) = 4 (2.925.5), P < 0.001].

### 3.4 Association between multiple HPV infections and cervical lesions above HSIL

**Table 3** presents the comparison of lesions more severe than HSIL between the p16(-) and p16(+) groups. Across all groups, our study found that compared to the p16(-) group, the p16(+) group had a higher association with cervical lesions worse than HSIL.

**Table 3:** Associations of HPV infection patterns and cervical lesion grades with p16.

Variables	Total	Event (%)	Crude		Adjusted	
			OR (95%CI)	P value	OR (95%CI)	P value
negative	870	125 (14.4)	1(Ref)		1(Ref)	
negative+	294	259 (88.1)	44.1 (29.55~65.84)	<0.001	43.06 (28.82~64.33)	<0.001
SH	759	99 (13)	0.89 (0.67~1.19)	0.439	0.88 (0.66~1.17)	0.371
SH+	676	636 (94.1)	94.76 (65.36~137.39)	<0.001	93.58 (64.47~135.85)	<0.001
MH	83	12 (14.5)	1.01 (0.53~1.91)	0.982	1 (0.53~1.9)	0.999
MH+	112	111 (99.1)	661.56 (91.54~4781.28)	<0.001	660.62 (91.39~4775.53)	<0.001
MO	550	75 (13.6)	0.94 (0.69~1.28)	0.7	0.93 (0.69~1.27)	0.67
MO+	446	416 (93.3)	82.65 (54.51~125.3)	<0.001	80.91 (53.28~122.87)	<0.001
other	996	148 (14.9)	1.04 (0.8~1.35)	0.764	1.03 (0.8~1.34)	0.794
other+	379	332 (87.6)	42.1 (29.39~60.31)	<0.001	41.54 (28.96~59.59)	<0.001

Models adjusted for age, gravidity, parity and cervical histology.

negative, HPV negative group; negative+, HPV negative group with p16(+); SH, Single HPV-16, 33, 52, or 58 infection; SH+, Single HPV-16, 33, 52, or 58 infection with p16(+); MH, multiple HPV-16, 33, 52, and 58 infection; MH+, multiple HPV-16; 33, 52, and 58 infection with p16(+).

MO, Multiple HPV infection with genotype other than HPV16, 33, 52, and 58. MO+, Multiple HPV infection with genotype other than HPV16, 33, 52, and 58 with p16(+). other, Single HPV infection with genotype other than HPV16, 33, 52, and 58. other+, Single HPV infection with genotype other than HPV16, 33, 52, and 58 with p16(+).

In the negative+ group, the risk of cervical lesions above HSIL was 43.06-fold higher than that of the negative group [adjusted OR=43.06, 95% CI: 28.8264.33, P<0.001]. In the SH+ group, the risk of cervical lesions above HSIL was 93.58-fold higher than that of the SH group [adjusted OR=93.58, 95% CI: 64.47135.85, P<0.001]. The MH+ group demonstrated the highest risk increase, with p16(+) patients having a 660-fold higher risk of cervical lesions above HSIL compared to the negative group [adjusted OR=660.62, 95% CI: 91.394775.53, P<0.001]. This group represented the most significant risk for cervical lesions above HSIL. In the other+ group, the risk of cervical lesions above HSIL was 41.54-fold higher than that of the other group [adjusted OR=41.54, 95% CI: 28.9659.59, P<0.001]. Similarly, in the MO+ group, the risk of lesions above HSIL was 80.91-fold higher than that of the other group [adjusted OR=80.91, 95% CI: 53.28~122.87, P<0.001].

## Discussions

Cervical cancer is unique as it is the only type of cancer with a clear etiology and complete tertiary prevention measures. The two most common ways of screening for cervical cancer are cervical cytology and HPV detection (13). Cytology is based on microscopic morphology and has limitations, such as complex grading, subjectivity, and variable diagnostic repeatability, leading to insufficient sensitivity. On the other hand, HPV tests have high sensitivity but lower specificity due to potential transient infections being missed, and they cannot reflect the extent or severity of HPV-induced lesions.

Countries with established cervical cancer screening programs are increasingly adopting HPV primary screening as the preferred method (14, 15). Early detection through improved screening methods can significantly improve survival rates for cervical cancer patients. Abnormal expression of p16 is closely related to HPV-16 and HPV-18 infections, and its expression increases with the progression of CIN and cervical cancer (16–18). Patients with p16-negative HPV-associated cervical cancer tend to have worse prognoses (19). Combining TCT with dual staining of p16/Ki67 has shown high sensitivity and specificity in detecting HSIL, making it an effective screening method (20).

Multiple infections are common in healthy women (15.8%) but less prevalent in cervical cancer patients (3%-4%), and the relationship between multiple infection and pathogenicity requires further study (18).

In our study, 5165 female patients with definite pathological diagnoses were included, with 1907 exhibiting positive p16 expression and 3258 showing negative p16 expression. P16 expression correlated positively with high-risk HPV types, including HPV-16, HPV-33, HPV-52, and HPV-58, with an increased risk compared to p16(-) cases (9).

The p16 gene, located on chromosome 9, encodes the p16 protein, which inhibits cell proliferation by preventing cells from entering the S phase (21, 22). Variations in the p16 gene and inactivation of its proteins are common in various malignant tumors, including cervical cancer (23).

Persistent infection with high-risk HPV is associated with cervical intraepithelial neoplasia and cervical cancer (24, 25). HPV can exist in free or integrated form, and persistent infection may lead to gene instability and lesion escalation (26, 27). The E7 gene of HPV inactivates the pRb protein, promoting cell cycle progression and potentially leading to feedback overexpression of p16 (22). Thus, the overexpression of p16 in tumor cells is linked to HPV infection (28).

Over 80% of patients with HPV infections experience transient infections, while 4% to 10% develop persistent HPV infections, leading to cervical lesions and potentially cancer (29). Among the 200 identified HPV types, HPV-16 and HPV-18 are the most common and pathogenic types (30). Multiple HPV infections are more common in LSIL and HSIL patients, with longer durations of infection increasing the risk of cervical lesions (30).

Positive p16 protein expression is correlated with increasing cervical lesion levels, making it a predictor of cervical lesion escalation (31, 32). Combining HPV with p16 testing can enhance cervical cancer detection and risk assessment (33). P16 expression has been proposed as a new indicator for cervical cancer screening (19).

The current study’s limitations include its retrospective cross-sectional design, which may introduce selection bias, and the potential impact of residual confounding factors. Multicenter prospective cohort studies are needed to validate the findings. Another potential limitation of this study is that heavy methylation of the p16 gene promoter region can lead to silencing and decreased expression of p16, resulting in false negative results by immunohistochemistry. In the latter study, it will be important to understand the potential confounding effects of high p16 methylation when interpreting p16 immunohistochemistry results in cervical specimens.

In conclusion, p16 expression is crucial in cervical lesion progression and is associated with high-risk HPV genotypes (HPV-16, 33, 52, and 58). Incorporating p16 testing into HPV screening can enhance cervical cancer detection. Further research on multiple HPV infections’ role in cervical lesion development will improve cervical cancer prevention and management.

## Data availability statement

The original contributions presented in the study are included in the article/supplementary material. Further inquiries can be directed to the corresponding authors.

## Ethics statement

The paper was approved by the Ethics Committee of Fujian Maternity and Child Health Hospital, Affiliated Hospital of Fujian Medical University (2022YJ002). The studies were conducted in accordance with the local legislation and institutional requirements. Informed consent was waived due to the retrospective nature of the study.

## Author contributions

YZ: Writing – original draft, Writing – review & editing. HBL: Writing – original draft, Writing – review & editing. XL: Data curation, Methodology, Writing – original draft, Writing – review & editing. ZL: Data curation, Writing – original draft, Writing – review & editing. QY: Data curation, Investigation, Writing – original draft. HWL: Data curation, Investigation, Writing – review & editing. ZZ: Data curation, Investigation, Writing – original draft. HY: Writing – review & editing. YS: Writing – review & editing. XZ: Writing – review & editing. YC: Writing – review & editing. JC: Writing – review & editing.
