# Corrigendum: Associations of multi-human papillomavirus infections with expression of p16 in a cohort of women who underwent colposcopy: a retrospective study of 5165 patients

**DOI:** 10.3389/fonc.2025.1570236

**Published:** 2025-03-07

**Authors:** Yulong Zhang, Haibo Li, Xiaowen Li, Zelong Li, Qianru You, Hanwen Liu, Zhiyan Zhao, Yanzhao Su, Xiangqin Zheng, Yusha Chen, Jiancui Chen, Huan Yi

**Affiliations:** ^1^ Department of Gynecology, Fujian Maternity and Child Health Hospital College of Clinical Medical for Obstetrics & Gynecology and Pediatrics, Fujian Medical University, Fuzhou, China; ^2^ Division of Birth Cohort Study, Fujian Maternity and Child Health Hospital, College of Clinical Medicine for Obstetrics & Gynecology and Pediatrics, Fujian Medical University, Fuzhou, China; ^3^ Integrated Biology, University of California, Berkeley, Berkeley, CA, United States; ^4^ Cervical Disease Diagnosis and Treatment Health Center, Fujian Maternity and Child Health Hospital College of Clinical Medical for Obstetrics & Gynecology and Pediatrics, Fujian Medical University, Fuzhou, China

**Keywords:** HPV, cervical cancer, p16, cervical lesions, retrospective study

In the published article, there was an error in the legend for **Figure 1** as published.

**Figure 1:**
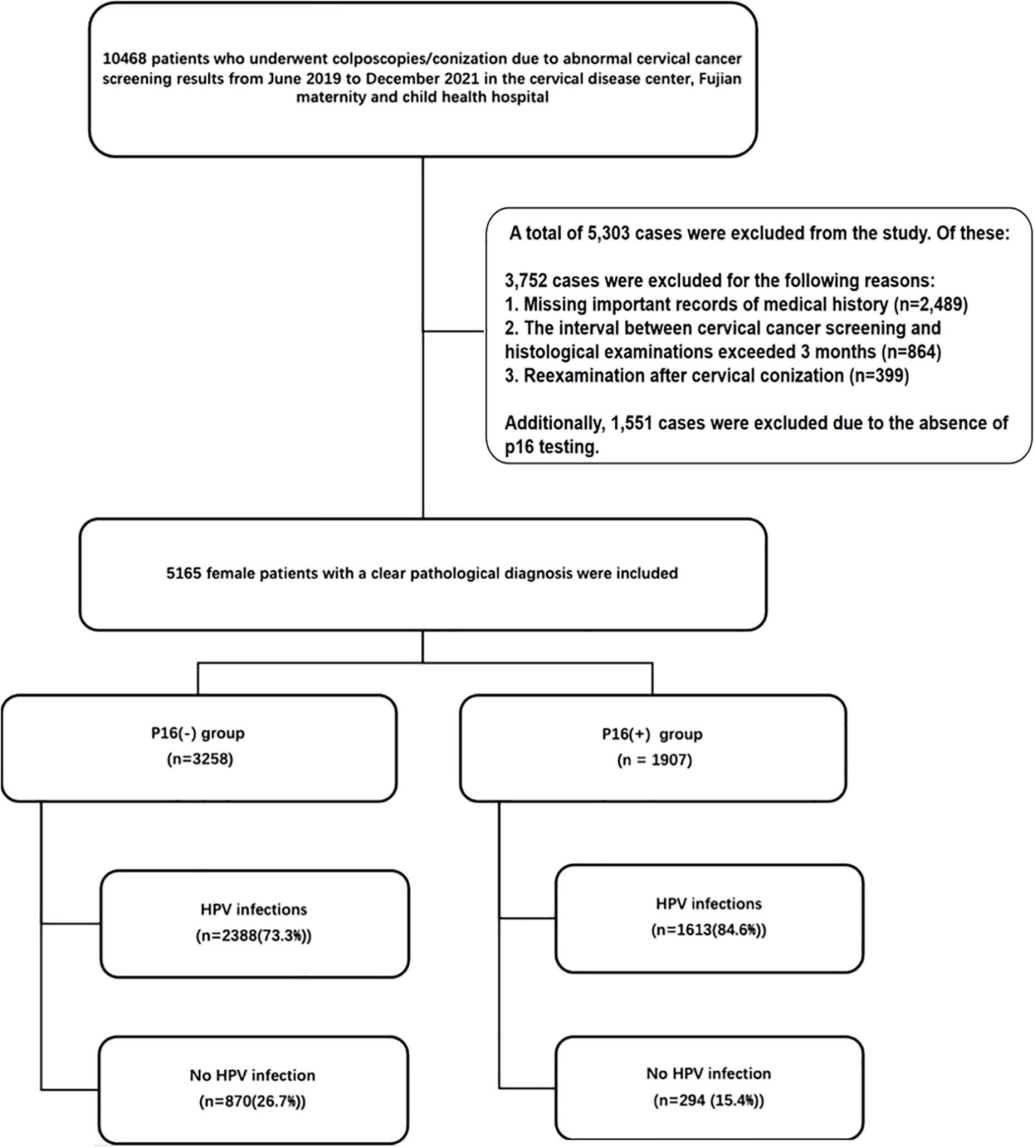
Out of a total of 10,468 patients who underwent colposcopies/conization due to abnormal cervical cancer screening results from June 2019 to December 2021, 5,303 cases were excluded. Of these, 3,752 cases were excluded for the following reasons: 1. Missing important records of medical history (n=2,489). 2. The interval between cervical cancer screening and histological examinations exceeded 3 months (n=864). 3. Reexamination after cervical conization (n=399). Additionally, 1,551 cases were excluded due to the absence of p16 testing. A total of 5,165 female patients with clear pathological diagnoses were included in the final analysis. HPV, human papillomavirus.

The corrected **Figure 1** legend appears below:

“Out of a total of 10,468 patients who underwent colposcopies/conization due to abnormal cervical cancer screening results from June 2019 to December 2021, 5,303 cases were excluded. Of these, 3,752 cases were excluded for the following reasons:

1. Missing important records of medical history (n=2,489).

2. The interval between cervical cancer screening and histological examinations exceeded 3 months (n=864).

3. Reexamination after cervical conization (n=399).

Additionally, 1,551 cases were excluded due to the absence of p16 testing.

A total of 5,165 female patients with clear pathological diagnoses were included in the final analysis. HPV, human papillomavirus.”

The authors apologize for this error and state that this does not change the scientific conclusions of the article in any way. The original article has been updated.

